# Phase II study of capecitabine and the oral mTOR inhibitor everolimus in patients with advanced pancreatic cancer

**DOI:** 10.1007/s00280-015-2730-y

**Published:** 2015-03-31

**Authors:** S. Kordes, H. J. Klümpen, M. J. Weterman, J. H. M. Schellens, D. J. Richel, J. W. Wilmink

**Affiliations:** 1 Department of Medical Oncology, Academic Medical Center Amsterdam, Meibergdreef 9, Amsterdam, The Netherlands; 2 Division of Clinical Pharmacology, Department of Medical Oncology, The Netherlands Cancer Institute, Plesmanlaan 121, Amsterdam, The Netherlands; 3 Department of Experimental Therapy, The Netherlands Cancer Institute, Plesmanlaan 121, Amsterdam, The Netherlands; 4 Division of Biomedical Analysis, Department of Pharmaceutical Sciences, Faculty of Science, University Utrecht, Universiteitsweg 99, Utrecht, The Netherlands

**Keywords:** mTOR, Pancreatic cancer, Capecitabine, Phase II, Targeted therapy, Everolimus

## Abstract

**Purpose:**

The combination of an mTOR inhibitor with 5-fluorouracil-based anticancer therapy is attractive because of preclinical evidence of synergy between these drugs. According to our phase I study, the combination of capecitabine and everolimus is safe and feasible, with potential activity in pancreatic cancer patients.

**Methods:**

Patients with advanced adenocarcinoma of the pancreas were enrolled. Eligible patients had a WHO performance status 0–2 and adequate hepatic and renal functions. The treatment regimen consisted of capecitabine 1000 mg/m^2^ BID day 1–14 and everolimus 10 mg daily (5 mg BID) in a continuous 21-day schedule. Tumor assessment was performed with CT-scan every three cycles. Primary endpoint was response rate (RR) according to RECIST 1.0. Secondary endpoints were progression-free survival, overall survival and 1-year survival rate.

**Results:**

In total, 31 patients were enrolled. Median (range) treatment duration with everolimus was 76 days (1–431). Principal grade 3/4 toxicities were hyperglycemia (45 %), hand-foot syndrome (16 %), diarrhea (6 %) and mucositis (3 %). Prominent grade 1/2 toxicities were anemia (81 %), rash (65 %), mucositis (58 %) and fatigue (55 %). RR was 6 %. Ten patients (32 %) had stable disease resulting in a disease control rate of 38 %. Median overall survival was 8.9 months (95 % CI 4.6–13.1). Progression-free survival was 3.6 months (95 % CI 1.9–5.3).

**Conclusions:**

The oral regimen with the combination of capecitabine and everolimus is a moderately active treatment for patients with advanced pancreatic cancer, with an acceptable toxicity profile at the applied dose level.

## Introduction

The PI3K/Akt pathway is an important intracellular signaling pathway that is often dysregulated in cancer [1]. Signal transduction of activated PI3K/Akt is transmitted through several downstream targets, including the mammalian target of rapamycin (mTOR) [2, 3]. Everolimus, an oral mTOR inhibitor, has demonstrated antitumor properties in vitro and in vivo including inhibition of cell proliferation, cell survival and angiogenesis and showed additive as well as synergistic effects when combined with other anticancer agents such as 5-fluorouracil (5-FU) [4–11].

Single-agent everolimus has been investigated in phase I–III clinical trials in patients with various types of advanced solid tumors [12–19]. These trials demonstrated that treatment with everolimus at 10 mg daily was well tolerated and showed clinical activity in some malignancies with an acceptable toxicity profile, consisting mainly of stomatitis and fatigue.

Clinical trials investigating everolimus in combination with other anticancer drugs have been performed or are ongoing, in short, confirming preclinical evidence that everolimus may be more efficacious when used in combination with other anticancer drugs [20–25]. Recently, a phase II study with the combination of low-dose capecitabine (650 mg/m^2^ BID day 1–14) and everolimus (5 mg BID) showed effectivity in heavily pretreated gastric cancer patients, and with an acceptable toxicity profile [26]. Previously, we demonstrated the safety and feasibility of the combination of capecitabine 1000 mg/m^2^ BID day 1–14 and continuous everolimus 5 mg BID in a 21-day schedule [27]. In this phase I study, two out of three patients with pancreatic cancer achieved a partial remission (PR).

In the pre-FOLFIRINOX era, gemcitabine was considered standard therapy for first-line treatment of pancreatic cancer patients, because of a significant improvement of the clinical benefit response, although the survival advantage was <1 month, in comparison with 5-FU [28, 29]. Although studies with the oral 5-FU analogue capecitabine in direct comparison with 5-FU or gemcitabine in patients with pancreatic cancer are not available, capecitabine seems to have comparable activity in this patient group [30].

In the present phase II study, we explored the activity of capecitabine and everolimus combination treatment as first- and second-line therapy in patients with advanced pancreatic cancer.

## Materials and methods

### Patient population

Eligible patients were aged ≥18 years, with histologically or cytologically confirmed advanced pancreatic cancer, and measurable lesions according to Response Evaluation Criteria in Solid Tumors (RECIST 1.0) [31]. Patients who had prior chemotherapy in the adjuvant setting or for metastatic disease were eligible. Adjuvant patients were considered second line if the chemotherapy free interval was <6 months before start study. Other eligibility criteria included WHO performance status ≤2, estimated life expectancy of ≥3 months, adequate bone marrow (white blood cell count ≥ 3.0 × 10^9^/L, platelets ≥ 100 × 10^9^/L) and adequate hepatic and renal function (serum bilirubin ≤ 1.5 × upper limit of normal (ULN), ALAT/ASAT ≤ 2.5 × ULN or in case of liver metastases ≤ 5 × ULN and serum creatinine ≤ 150 μmol/L). Patients were ineligible if they had established alcohol abuse, drug addiction and/or psychotic disorders that made adequate follow-up unlikely. Women who were pregnant or lactating were also excluded. All patients gave written informed consent. The study was approved by the Medical Ethics Committee of the Academic Medical Center Amsterdam and was conducted in accordance with the Declaration of Helsinki and Good Clinical Practice guidelines. The trial was registered online (ClinicalTrials.gov identifier: NCT01079702).

### Study design and treatment

This was a phase II, open-label, single-center study to assess the antitumor activity and safety of the combination of everolimus and capecitabine. The primary endpoint was response rate. The study was conducted at the Amsterdam Medical Center, The Netherlands. Everolimus was administered continuously at an oral dose of 5 mg BID. The first 7 days of treatment patients were treated with single-agent everolimus to reach steady-state drug concentrations. Treatment with capecitabine 1000 mg/m^2^ BID started on day 8 and was given twice daily for 14 days in a three weekly cycle. Toxicity was graded using the National Cancer Institute Common Terminology Criteria for Adverse Events (CTCAE) version 3.0 and was assessed every treatment cycle [32]. Tumor measurements were performed at baseline and every three cycles, and responses were evaluated in accordance with RECIST 1.0 [31]. Patients continued treatment until disease progression, withdrawal of consent or in case of intolerability. Overall survival was defined from start of study treatment until death. Progression-free survival was defined from start of study to clinical or radiological progression or death. When treatment was discontinued due to other reasons, date of last documented assessment was used and censored.

### Statistical analysis

For sample size calculations, the two-stage design according to Gehan for estimating the response rate was used [33]. In the first stage, 14 patients were entered. If no responses were observed in the first stage, then the trial would be terminated because the absence of response (0/14) has a probability <0.05 if the true response rate is 0.20. We choose for an estimate with approximately a 10 % standard error, with an accrual of 11 patients in the second stage. For the evaluation of the safety, efficacy parameters descriptive statistics were applied using SPSS statistics. Intention to treat analysis was used.

## Results

In total, 31 patients with advanced pancreatic cancer were enrolled between June 2010 and November 2011. After a partial response in the first stage of 14 patients, 11 patients were enrolled in the second stage. Six additional patients were enrolled to ensure 25 patients receiving at least one full cycle of the treatment combination for a complete safety analysis (see below).

Patient characteristics are listed in Table 1. The majority of patients had metastatic adenocarcinoma and a WHO performance status of 0–1. The study group consisted of 15 first-line patients and 16 second-line patients. Eighteen patients received prior gemcitabine-based chemotherapy; 11 in palliative setting, six adjuvant (five patients with chemotherapy free interval <6 months) and 1 as part of neoadjuvant chemoradiotherapy.

**Table 1:** Patient characteristics

	*N*	%
No. of patients	31	
Gender		
Male	15	48
Female	16	52
Race		
Caucasian	30	97
Asian	1	3
Median age (years)	63	
Range	37–77	
Stage		
Metastatic	29	94
Locally advanced	2	6
WHO performance status		
0	16	52
1	11	35
2	4	13
Histology		
Adenocarcinoma	30	97
Acinar cell carcinoma	1	3
Prior therapy		
First line	15	48
Neoadjuvant CRT	1	3
Adjuvant gemcitabine	1	3
Second line	16	52
Adjuvant gemcitabine^a^	5	16
Palliative	11	35

*WHO* World Health Organization, *CRT* chemoradiotherapy

^a^Progression during or within 6 months after adjuvant treatment

Overall, a total of 147 treatment cycles were given, with a median (range) of 3 (1–9) cycles per patient. Median (range) treatment duration with everolimus was 76 (1–431) days (Table 2). Six patients temporarily interrupted treatment with everolimus due to adverse events. Following treatment interruption, five patients received a 50 % dose reduction of everolimus and the other continued treatment at the full dose of everolimus. Due to adverse events, treatment with capecitabine was interrupted in 15 patients resulting in dose reductions for capecitabine in 14 patients.

**Table 2:** Treatment administration of the combination of everolimus and capecitabine

*Evaluable patients (N* *=* *31)*	
No. of treatment cycles	
Median	3
Range	1–15
No. of treatment days with everolimus	
Mean ± SD	104 ± 93
Median	76
Range	1–431
*Everolimus dose delivery*	
No. of patients (%)	
Dose reduction due to toxicity	5 (16)
Temporary treatment disruption due to toxicity	6 (19)
*Capecitabine dose delivery*	
No. of patients (%)	
Dose reduction due to toxicity	14 (45)
Temporary treatment disruption due to toxicity	15 (48)

*SD* standard deviation

### Safety

Six patients did not receive a complete cycle of the treatment combination; two patients refusal, three patients had early clinical progression, and one patient died of non-treatment-related septic cholangitis. Treatment-related toxicities of these patients were included in the intention to treat toxicity analysis.

Table 3 lists the treatment-related CTC grade 1–2 and grade 3–4 adverse events. The most frequently reported clinical toxicities of any grade included mucositis, skin reactions, fatigue, nausea and diarrhea. Severe clinical toxicities were not frequent. Grade 3–4 hand-foot syndrome was observed in five patients, diarrhea in two patients, stomatitis, skin rash and vomiting in one patient. Three patients developed grade 3 hematological toxicity (thrombocytopenia and anemia). Hyperglycemia of any grade was the most frequently reported biochemical toxicity, resulting in clinical relevance (grade 3) in fourteen patients. Grade 3 levels of alkaline phosphatase, hypokalemia and hyperbilirubinemia occurred in two, five and one patients, respectively.

**Table 3:** Treatment-related grade 1–2 and grade 3–4 adverse events

	Total [*n* (%)]	
No. of patients	*N* = 31 (100)	
CTC grade	Grade 1–2	Grade 3–4
*Adverse event*		
Mucositis^a^	18 (58)	1 (3)
Fatigue	17 (55)	
Hand-foot syndrome	9 (29)	5 (16)
Diarrhea	13 (42)	2 (6)
Nausea	16 (52)	
Skin^b^	20 (65)	1 (3)
Anorexia	8 (26)	
Vomiting	12 (39)	1 (3)
Neuropathy	7 (23)	
Constipation	4 (13)	
Ankle edema	5 (16)	
Epistaxis	4 (13)	
Infection	3 (10)	
*Hematology*		
Anemia	25 (81)	1 (3)
Thrombocytopenia	14 (45)	2 (6)
Neutropenia	12 (39)	
*Clinical chemistry*		
Hyperglycemia^c^	13 (42)	14 (45)
AP	19 (61)	2 (6)
ASAT	11 (35)	
ALAT	12 (39)	
Hypokalemia	10 (32)	5 (16)
Hyponatremia	7 (23)	
Hypertriglyceridemia	7 (23)	
GGT	13 (42)	9 (29)
Bilirubin	4 (13)	1 (3)
Hypercholesteremia	3 (10)	

*GGT* gamma-glutamyltransferase, *ASAT* aspartate aminotransferase, *ALAT* alanine aminotransferase, *AP* alkaline phosphatase

^a^Mucositis including aphthous ulcers and stomatitis

^b^Skin toxicity includes rash, itching, color and nail changes

^c^Non-fasting glucose

### Antitumor activity

A total of 31 participating patients had measurable disease according to RECIST 1.0 at start of treatment. Nine of these 31 patients were not available for radiological response evaluation due to early discontinuation of the study medication prior to the first planned radiological evaluation time point and were considered as progressive. Two patients had a partial response (6.5 %). Ten patients had stable disease (32 %), and nineteen patients were progressive (61 %) (Table 4).

**Table 4:** Response rates for the entire cohort (*N* = 31)

Type of response	Entire cohort (*n* = 31)		First line (*n* = 15)		Second line (*n* = 16)	
PR	2	65 %	2	13 %	0	0 %
SD	10	32 %	8	53 %	2	13 %
PD	19	61 %	5	33 %	14	87 %

Per protocol radiological response evaluable patients (*N* = 22). Nine patients discontinued the study before radiological tumor response evaluation was performed (PD)

*PD* progressive disease, *PR* partial remission, *SD* stable disease

**Fig. 1:**
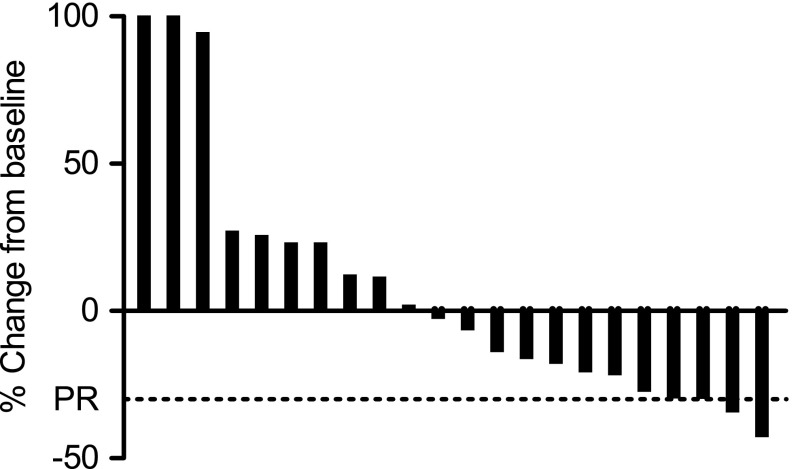
Best confirmed change from baseline in sum of longest diameters of target lesion size (%), by RECIST 1.0

In first-line patients, PR and SD were seen in 2 (13 %) and 8 (53 %), respectively. In second-line patients, no patients had PR and 2 (13 %) patients had SD. The waterfall plot shows radiologic responses for all radiologic evaluable patients (Fig. 1).

Among the nine patients who discontinued treatment early and who were not available for the first radiological response evaluation, two patients refused further therapy, three patients had clinical progressive disease and one patient developed grade 3 diarrhea and had deterioration of the performance status. Three patients died before radiological evaluation, two patients due to tumor progression and one patient died of non-treatment-related septic cholangitis during the first cycle

**Fig. 2:**
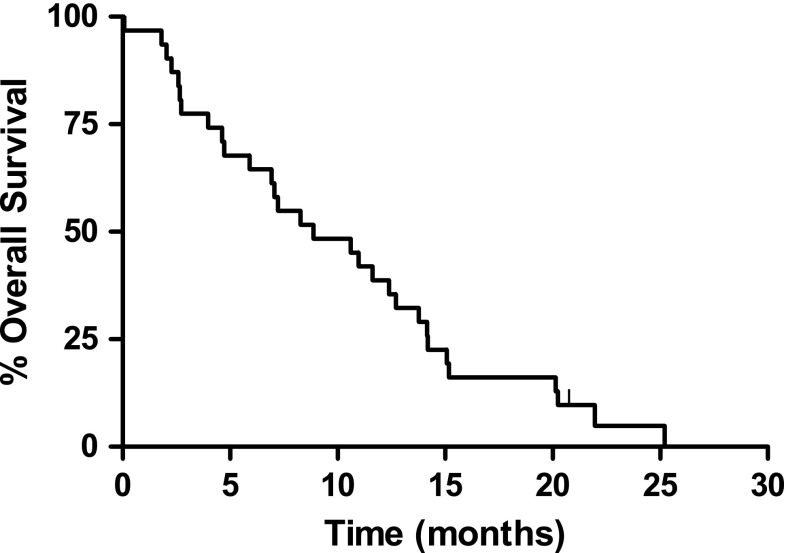
Kaplan–Meier curve of overall survival

At the time of the intention to treat analysis, one patient was still alive. For the entire cohort, the one-year survival rate was 38.7 % and the median overall survival was 8.9 months (95 % CI 4.6–13.1 months) (Fig. 2). Overall survival was 12.4 months (95 % CI 10.2–14.6) and 5.9 months (95 % CI 1.6–10.2) in first (*n* = 15)- and second-line patients (*n* = 16), respectively. Median progression-free survival (PFS) was 3.6 months (95 % CI 1.9–5.3), 5.7 months (95 % CI 2.0–9.5) and 2.3 months (95 % CI 2.2–2.5) for all, first- and second-line patients, respectively.

## Discussion

In this phase II study, exploring the combination of capecitabine with the oral mTOR inhibitor everolimus in patients with advanced pancreatic cancer moderate treatment activity was observed with a response rate (RR) of 6.5 % and an overall survival (OS) of 8.9 months.

As a single-arm phase II study, the contribution of everolimus in this treatment regimen is difficult to determine. However, our results can be compared with previous studies using capecitabine as monotherapy. A RR of 7.1 % was seen in a study of capecitabine (1250 mg/m^2^ BID) in 42 patients with advanced pancreatic cancer as first-line treatment [30]. A randomized phase II study with pre-treated pancreatic cancer patients showed a RR of 9.4 % for the 32 patients in the capecitabine (1250 mg/m^2^ BID) control arm [34].

The endpoint OS in non-randomized phase II studies, should be used with care, because of the constraints of historical control groups and inadequate sample sizes, especially in subgroup analyses. Nevertheless, the median OS of 8.9 months in the entire cohort, 12.4 months in first-line patients and 5.9 months in second-line patients reported in this study are encouraging. The overall survival for capecitabine monotherapy in first-line patients reported by Cartwright et al. [30] was 5.9 months (95 % CI 2.8–9.0 months). And OS in second-line patients, reported by Bodoky et al. [34] was 5.0 months. Therefore, the addition of everolimus to capecitabine might enhance efficacy of capecitabine monotherapy, especially in first-line patients.

The most commonly reported treatment-related clinical side effects were stomatitis, fatigue and hand-foot syndrome. Although stomatitis is a common adverse event of both capecitabine and everolimus as single agent, this overlapping toxicity remained mild to moderate in severity in this study and led to dose reduction in only one patient. We assume at most marginally additive toxicity of capecitabine to everolimus since the frequency of stomatitis in this study was similar to that in studies with single-agent everolimus [30, 34, 35]. The frequency of fatigue was increased compared with single-agent studies of either agent, suggesting an additive toxic effect of the combination. An alternative explanation for the increased incidence of fatigue might be disease related. Patients with pancreatic cancer have a high probability to develop disease-related fatigue [36]. Hand-foot syndrome can be solely attributed to capecitabine, since this has not been observed before in single-agent everolimus trials. This well-known side effect of capecitabine resulted in dose reductions of capecitabine in 45 % of the patients, which is only 10 % higher than in a previous study with colorectal cancer patients receiving capecitabine monotherapy and might be related to the combinatory effects of the study drugs [37]. Grade 3 hyperglycemia was seen in 45 % of the patients in the non-fasting blood draws, which is more than seen in everolimus monotherapy in this patient group (18 %), but it mostly remained at manageable levels and did not lead to dose reductions [14]. Other adverse events included diarrhea, anorexia, taste loss, neuropathy and skin rash, but remained non-severe. The frequency and severity of toxicity confirmed the data from our previous phase I study [27].

In contrast to the outlined acceptable toxicity findings, a prior phase I study that combined the mTOR inhibitor temsirolimus and 5-FU demonstrated dose limiting stomatitis and hematological toxicity, and in the expansion cohort, two patients died due to mucositis with bowel perforation [38]. An explanation for this difference could be dose related. The dose of temsirolimus in the expansion cohort of that phase I study was 45 mg/m^2^ per week, while the recommended dose used as monotherapy in renal cell carcinoma is 25 mg per week. The phase II study of capecitabine (650 mg/m^2^ BID) and everolimus (5 mg BID) in gastric cancer patients, showed less toxicity, especially in all grade hand-foot syndrome (35 vs 13 %), fatigue (55 vs 6 %) and diarrhea (48 vs 22 %) [26]. This is probably due to the lower dose of capecitabine and the different disease.

Recently, the combination of conventional chemotherapy (gemcitabine) with everolimus is examined as first-line treatment for pancreatic cancer patients [39]. Everolimus 5 mg BID could not be combined with full-dose gemcitabine, as the maximum tolerated dose was already met at 400 mg/m^2^ [39]. In comparison with the present study, this might indicate that for combining everolimus with conventional chemotherapy, capecitabine is easier to administer at therapeutical doses.

In conclusion, we showed that continuous everolimus 5 mg BID combined with capecitabine 1000 mg/m^2^ for 14 days every 3 weeks is a feasible and moderately efficacious outpatient oral treatment regimen, but only in first-line pancreatic cancer patients.
